# Nanomagnetic Actuation of Hybrid Stents for Hyperthermia Treatment of Hollow Organ Tumors

**DOI:** 10.3390/nano11030618

**Published:** 2021-03-02

**Authors:** Benedikt Mues, Benedict Bauer, Anjali A. Roeth, Jeanette Ortega, Eva Miriam Buhl, Patricia Radon, Frank Wiekhorst, Thomas Gries, Thomas Schmitz-Rode, Ioana Slabu

**Affiliations:** 1 Institute of Applied Medical Engineering, Helmholtz Institute, Medical Faculty, RWTH Aachen University, Pauwelsstraße 20, 52074 Aachen, Germany; mues@ame.rwth-aachen.de (B.M.); smiro@ame.rwth-aachen.de (T.S.-R.); 2 Institut für Textiltechnik, RWTH Aachen University, Otto-Blumenthal-Straße 1, 52074 Aachen, Germany; benedict.bauer@ita.rwth-aachen.de (B.B.); jeanette.ortega@ita.rwth-aachen.de (J.O.); thomas.gries@ita.rwth-aachen.de (T.G.); 3 Department of General, Visceral and Transplant Surgery, RWTH Aachen University Hospital, Pauwelsstraße 30, 52074 Aachen, Germany; aroeth@ukaachen.de; 4 Institute of Pathology, Electron Microscopy Facility, RWTH University Hospital Aachen, Pauwelsstraße 30, 52074 Aachen, Germany; ebuhl@ukaachen.de; 5 Physikalisch-Technische Bundesanstalt, Abbestraße 2-12, 10587 Berlin, Germany; patricia.radon@ptb.de (P.R.); Frank.Wiekhorst@ptb.de (F.W.)

**Keywords:** magnetic nanoparticles, hyperthermia efficiency, hybrid implants, Brownian relaxation, Néel relaxation, stents, tumor therapy

## Abstract

This paper describes a magnetic nanotechnology that locally enables hyperthermia treatment of hollow organ tumors by using polymer hybrid stents with incorporated magnetic nanoparticles (MNP). The hybrid stents are implanted and activated in an alternating magnetic field to generate therapeutically effective heat, thereby destroying the tumor. Here, we demonstrate the feasibility of nanomagnetic actuation of three prototype hybrid stents for hyperthermia treatment of hollow organ tumors. The results show that the heating efficiency of stent filaments increases with frequency from approximately 60 W/g_Fe_ (95 kHz) to approximately 250 W/g_Fe_ (270 kHz). The same trend is observed for the variation of magnetic field amplitude; however, heating efficiency saturates at approximately 30 kA/m. MNP immobilization strongly influences heating efficiency showing a relative difference in heating output of up to 60% compared to that of freely dispersed MNP. The stents showed uniformly distributed heat on their surface reaching therapeutically effective temperatures of 43 °C and were tested in an explanted pig bile duct for their biological safety. Nanomagnetic actuation of hybrid stents opens new possibilities in cancer treatment of hollow organ tumors.

## 1. Introduction

For patients with tumors of endoluminal organs, e.g., tracheobronchial carcinoma, esophagus adenocarcinoma, or bile duct Klatskin tumors, life-threatening situations arise due to tumor ingrowth and thereby occlusion of the hollow organ [1,2,3,4]. In approximately 20% of the cases, such tumors are non-resectable, and treatments are restricted to implantation of metallic or plastic stents to widen the occluded endoluminal site [5,6]. However, usually due to tumor tissue ingrowth, a narrowing of the hollow organs, so-called restenosis or reocclusion, develops [7,8]. In order to prevent this, magnetic fluid hyperthermia (MFH) treatment can be employed. In this way, therapeutically effective heat with temperatures in a range of 42 to 46 °C is generated, destroying tumor cells via apoptosis [9,10]. Magnetic nanoparticles (MNP) are known as promising heating agents suitable for the design of hybrid materials and textile implants with controllable heat output and specific saturation temperatures and, therefore, are widely investigated for cancer treatment by MFH. MNP have been also be used in tumor treatment by photothermia due to their ability to convert the energy of absorbed light into heat [11,12]. As heat enhances tumor sensitivity, MFH was also investigated in combination with other cancer treatments such as hadron therapy [13] or immune therapy [14]. The technology of nanoparticle incorporation into textiles enhancing their functionality such as UV resistance, antimicrobial properties, or magnetic guidance was reported previously [15,16,17]. MNP inside implant materials were used before as contrast agents to visualize implant structure and functionality with magnetic resonance imaging (MRI) [18,19,20]. Such engineered materials loaded with MNP are also known as magnetic scaffolds. Biodegradable magnetic scaffolds made of polymers such as chitosan–glycerophosphate, poly(L-lactic acid) (PLLA), or poly-caprolactone (PCL) have been widely investigated in tissue engineering for nerve regeneration [21], improvement of wound healing after surgical resection of a malignant skin tumor [22], and design of cardiac tissue [23]. Fibrous magnetic scaffolds are particularly suitable for designing smart hyperthermia nanofiber platforms to combine drug and heat release [24,25,26,27]. In the case of bone tissue engineering, magnetic scaffolds of biodegradable polymers (e.g., PCL, gelatin) and ceramics (e.g., hydroxyapatite, akermanite) have been investigated to avoid recurrence of bone tumors after surgery by local hyperthermia treatment [28,29,30]. In this way, a temperature increase of 20 °C was achieved, resulting in a survival fraction of only 30% of VX2 cancer cells after treatment [31].

To enable MFH, the MNP are excited in an alternating magnetic field (AFM), transforming its energy into heat via magnetic relaxation processes (Néel and Brownian relaxation). To facilitate local MFH treatment on cellular level, MNP are usually directly injected into the tumor [32] or accumulated at the tumor site via magnetic targeting [33]. There, MNP interactions with cancer cells play an important role regarding their heat release inside the cell [34]. Recently, it was shown that MNP binding to the cell surface and internalization of bigger MNP agglomerates inside the cells leads to a loss in MNP heating efficiency, especially because of the absence of Brownian relaxation contributions to the overall magnetic relaxation process [35,36]. MNP agglomeration was demonstrated to have an enhancing effect, while MNP immobilization was shown to cause a drop in heating efficiency [37,38]. Along the same lines, gradual immobilization of MNP inside hydrogels led to a loss in heating efficiency by one third [39]. These characteristics of heating performance are a result of a complex interplay between field parameters, MNP immobilization, and agglomeration. There are two established MNP heating theories, linear response theory (LRT) and Stoner–Wohlfarth model based theory (SWMBT), describing the MFH physics [40]. However, they lack comprehensive implementation of MNP dynamic relaxation processes. To account for the interaction phenomena and the absence of the Brownian relaxation in magnetic scaffolds, the so-called Cole–Cole model, an extension of the Debye model used in the LRT, was suggested for the description of the relaxation dynamics [41,42,43]. With this model, the temperature increase of magnetic scaffolds activated in an AMF was explained based on a smooth decrease of MNP susceptibility for increasing frequency. This differs from the expectations for dispersed MNP, which exhibit a peak susceptibility as described with the Debye model [41,44]. A similar change of the susceptibility was also observed for MNP embedded in hydrogel [45]. Further, advanced stochastic non-equilibrium magnetic relaxation simulations provided a deeper understanding of the fundamentals of MNP magnetic relaxation processes in AMF [46,47]. In such simulations, the heating efficiency was determined in dependency of MNP magnetic size and anisotropy energy constants as well as of the AFM parameters. In Monte-Carlo simulations, the effects of MNP immobilization and interactions on MFH were also investigated [48].

Similar to magnetic scaffolds, immobilization and agglomeration of MNP inside hybrid materials and filaments used for the design of implants change their heating properties. The presence of MNP agglomerates inside filaments after a melt-spinning process was reported before [18]. In this case, MNP are fully immobilized, and Brownian rotation can be considered as blocked. In order to optimize hyperthermia performance of hybrid implants, AMF parameters (amplitude, frequency) must be adjusted with respect to MNP properties (size, magnetization) so that the contribution of Néel relaxation to the heating efficiency is maximized. According to previous reports, for a Néel dominated magnetic relaxation, small MNP sizes (<20 nm) in combination with an AMF frequency of about 100 kHz are most suitable parameters to enhance the MFH performance [49,50,51].

For MFH treatment of hollow organ tumors, we developed hybrid polypropylene (PP) stents with incorporated magnetic nanoparticles (MNP), to enable local heat by their nanomagnetic actuation in an AMF. A similar approach of tumor treatment by heat induction in metallic stents was investigated before; however, the energy uptake from the AMF was not sufficient, and the control of temperature was difficult, resulting in unintended extent of necrosis [52,53]. Compared to previous works on magnetic scaffolds, we present a non-degradable prototype polymer stent combining local hyperthermia and mechanical widening of hollow organs. In the present work, we evaluate the heating performance of hybrid filaments and braided stents with three different MNP concentrations depending on various AMF parameters. The heating performance at three different frequencies (95, 140, and 270 kHz) and at different magnetic field amplitudes varying from 14 to 65 kA/m is determined. MNP are thoroughly characterized, and the relevant properties are discussed, especially regarding the MNP agglomeration and immobilization inside the filaments as well as their magnetic relaxation in static and alternating magnetic fields. Finally, the feasibility of achieving therapeutic temperatures of approximately 43 °C at the surface of three prototype stents designed for the bile duct is demonstrated, and the biological safety of such a stent placed in a porcine bile duct is evaluated by histological examination.

## 2. Materials and Methods

### 2.1. Materials

For the synthesis of the MNP, the chemicals iron(III) chloride hexahydrate (FeCl_3_·6H_2_O, p.a., ≥99%, Sigma-Aldrich, Darmstadt, Germany), iron(II) chloride tetrahydrate (FeCl_2_·4H_2_O, p.a., ≥98%, Sigma-Aldrich, Darmstadt, Germany), ammonium hydroxide (NH_4_OH, ACS reag., 28–30%, Sigma-Aldrich, Darmstadt, Germany), and lauric acid (C_12_H_24_O_2_ > 98%, FCC, Sigma-Aldrich, Darmstadt, Germany) were used. The chemicals acrylamide (Aam, ≥99%, Sigma-Aldrich, Darmstadt, Germany), N,N′-methylenebisacrylamide (BIS, 99%, Sigma-Aldrich, Darmstadt, Germany), ammonium peroxodisulfate (APS, ≥98%, AppliChem, Darmstadt, Germany), and N,N,N′,N’-tetramethylethylene-diamine (TEMED, 99%, Sigma-Aldrich, Darmstadt, Germany) were used for the synthesis of the acrylamide hydrogel. Technical grade polypropylene (PP) pellets (Moplen HP561R) were purchased from LyondellBasell Industries N.V. (Rotterdam, Netherlands). Agarose (Carl Roth, Karlsruhe, Germany) was used to prepare a tissue-like gel environment in which the inductive heating experiments were carried out. For the cytotoxicity tests, RPMI cell culture medium (21875-034, Life Technologies, Carlsbad, CA, USA) and CellTiter-Glo 2.0 assay (G7570, Promega, Madison, WI, USA) were used.

### 2.2. Synthesis of MNP

First, lauric acid coated iron oxide magnetite (Fe_3_O_4_) nanoparticles were synthesized via the co-precipitation of Fe^2+^ and Fe^3+^ ions (molar ratio Fe^2+^/Fe^3+^ of 1/2) in the presence of a base as described in [54]. Briefly, 16 g FeCl_3_ and 8 g FeCl_2_ were dissolved in 67.4 mL deionized water. Subsequently, 33.4 mL of NH_4_OH were added dropwise under constant stirring at room temperature. The precipitated MNP were washed several times with 100 mL of a 0.7 M NH_4_OH solution to remove the chloride ions. For the coating, the resulting MNP suspension was heated up in an oil bath at 90 °C, and 3.0 g lauric acid was added under constant stirring. The MNP suspension was cooled down to room temperature and centrifuged for 15 min at 4000 rpm with a Rotina 420R Centrifuge (Thermo Scientific Inc., Waltham, MA, USA). Subsequently, the in water dispersed MNP (in the following denoted as MNP disp) were freeze-dried at −85 °C and 0.04 mbar in a lyophilisator (Alpha 2-4 LDplus, Martin Christ Gefriertrocknungsanlagen GmbH, Osterode, Germany) for at least 12 h, resulting in an MNP powder.

### 2.3. Synthesis of Acrylamide Hydrogels with MNP

The MNP were immobilized inside acrylamide hydrogels (in the following denoted as MNP immob) with an adjustable mesh size as described in literature [39]. For the preparation of a hydrogel with a mean mesh size of approximately 10 nm, a polymer volume fraction of *ν*_pol_ = (*ρ^−^*^1^_Aam_(*m*_Aam_ + *m*_BIS_))/(*ρ*^−1^_H2O_*m*_tot_) = 8% and a crosslinker mole fraction of α = *n*^−1^_BIS_(*n*_BIS_ + *n*_Aam_) = 1% were used. Here, *ρ*_Aam_ = 1.3 g·cm^−3^ and *ρ*_H2O_ = 1.0 g·cm^−3^ denote the density of polyacrylamide and deionized water, respectively [55]. Further, *m*_Aam_, *m*_BIS_, *m*_tot_, are the masses of Aam, BIS, and the mass of total solution, while *n*_BIS_ and *n*_Aam_ denote the amount of substance of BIS and Aam. For the preparation, Aam and BIS were completely dissolved in water. Subsequently lauric acid stabilized MNP were added to the solution and vortexed several times. In order to initialize and catalyze the free radical polymerization, mass fractions 0.2% of APS as well as TEMED were added to the solution, respectively. The polymerization was kept for 2 h at room temperature. All amounts were selected to reach a resulting sample volume of 1 mL with an iron concentration of 1.1 mg(Fe)/mL for the purposes of comparability.

### 2.4. Production of PP Filaments and Stents

Three different hybrid filaments were produced by melt-spinning of PP pellets with 3%(*w/w*), 5%(*w/w*), and 7%(*w/w*) freeze-dried MNP (in the following denoted as PP@3%MNP, PP@5%MNP, and PP@7%MNP). For that, PP pellets and MNP were melt-spun at 230 °C and processed with a twin-screw extruder (MC 15 Micro Compounder, Xplore Instruments BV, Sittard, Netherlands), forming a homogeneous mixture that was pressed through a nozzle. Subsequently, 30 cm of the hybrid filaments PP@3%MNP, PP@5%MNP, and PP@7%MNP were braided into stents (in the following denoted as St@3%MNP, St@5%MNP, and St@7%MNP) and heat set at 140 °C for 30 min.

### 2.5. Transmission Electron Microscopy (TEM)

The core diameters *d*_core_ of the MNP and the MNP agglomerates inside the PP filaments were investigated via TEM with a Zeiss LEO 906 microscope (Carl Zeiss GmbH, Oberkochen, Germany) operated at 60 kV. For the dispersed MNP, 1 µL of the sample was pipetted on formvar-carbon-coated nickel grids (200 mesh) (Electron Microscopy Sciences, Hatfield, PA, USA) and air-dried under ambient conditions. For the spherical shaped MNP, the core diameter *d* of about 150 particles was measured using the software GIMP and fitted with the cumulative log-normal distribution probability density function (CDF) from which the mean and variance are calculated (see Supplementary Materials S1). The PP filaments were embedded in epon resin, cut into thin slices using a diamond cutter, and placed on nickel grids. For the evaluation of ellipsoidal MNP agglomerates inside the PP filaments, both the major and the minor axis of about 150 agglomerates were measured. The mean core or agglomerate size was obtained by fitting the CDF to the measured data.

### 2.6. Dynamic Light Scattering (DLS)

The hydrodynamic diameter *d*_h_ of MNP dispersed in deionized H_2_O was measured by DLS using a Zetasizer Nano S (Malvern Instruments Ltd., Worcestershire, UK) at a wavelength of *λ* = 633 nm (detection angle of 173°). All measurements were performed at 20 °C and repeated a total of three times. The mean and variance of the hydrodynamic size are obtained by fitting the log-normal distribution probability density function (PDF) to the measured intensity data (see Supplementary Materials S1).

### 2.7. X-ray Diffraction (XRD)

To investigate the as synthesized MNP crystal structure and crystalline size *d*_cryst_, XRD measurements were performed. For this, an X’pert diffractometer (PANalytical B.V., Almelo, Netherlands) was operated at a wavelength of *λ*_Kα_ = 1.5 Å of a copper anode and an acceleration current of *I* = 40 kA. For the preparation, a glass substrate, which was cleaned in acetone and isopropanol, was covered with 100 µL 0.1% poly-L-lysine. Dispersed MNP were pipetted on the substrate and air-dried under ambient conditions. Grazing incident measurements were performed with a constant incident angle *ω* = 0.7°, and the diffracted signal was recorded in increments of 0.02° over a rage of 2*θ* = (20°–70°). The XRD measurements for the MNP resulted in an intensity profile of the diffracted X-rays. The Bragg angles were determined using the pseudo-Voigt function as a fitting model [56] (see Supplementary Materials S1). Further, the MNP crystalline size *d*_cryst_ was determined using the modified Scherrer equation [57] (see Supplementary Materials S1).

### 2.8. Iron Concentration Determination

The iron concentration inside the PP filaments was examined by thermogravimetric analysis (TGA) using a STARe System TGA/DSC1 (Mettler Toledo GmbH, Gießen, Germany) and by photometric absorption (PA) measurements using a Spectrometer Ultrospec 2100 pro (Biochrom Ltd., Cambridge United Kingdom). For the TGA measurements, the samples were continuously heated (10 °C/min) up to 800 °C until the polymer and MNP coating degraded completely. The weight of the residue was allocated to that of iron oxide (magnetite Fe_3_O_4_, see Section 3 Results and Discussion), from which the iron concentration inside the filament was deduced. For PA measurements, the filaments were first treated with HCl and HNO_3_ to dissolve the iron ions, and the iron concentration in the solution was determined based on complexation of Fe^3+^ with Tiron (4,5-Dihydroxy-1,3-benzenedisulfonic acid disodium salt monohydrate) [58]. TGA and PA measurement results were used to calculate the weighted average of the iron concentration inside the filaments (see Supplementary Materials S2 Table S2).

### 2.9. Cytotoxicity Tests of Hybrid Filaments

As the hybrid stents are designed for the implantion in patients, it must be ensured that the filaments are not toxic to cells and do not cause cell damage. Therefore, the toxic effect on cells, so-called cytotoxicity, of the filaments PP@3%MNP, PP@5%MNP, and PP@7%MNP was tested using the mouse fibroblast cell line L929 from the German Collection of Microorganisms and Cell-Cultures. This cell line was chosen according to ISO 10993-5, as it is sensitive towards toxic effects. It is, therefore, used for cytotoxic testing, independent from the target tissue [59,60,61,62,63,64]. For pretreatment, the cells were seeded with Roswell Park Memorial Institute (RPMI) medium with 10%(*v/v*) fetal calf serum (FCS) and 1%(*v/v*) Penicillin/Streptomycin. Approximately 0.5 mg of each filament was disinfected and magnetically fixed on the ground of a well. After that, a cell suspension with 10,000 L929 cells in RPMI medium was added to the filaments and incubated at 37 °C and 5% CO_2_ for 24 h. Then, the supernatant was removed, and equal amount of RPMI medium as well as CellTiter-Glo were added. The CellTiter-Glo assay is based on the quantification of ATP produced by metabolically active cells. After cell lysis, a luminescent signal is generated, which is proportional to the amount of ATP and is recorded at an integration time of 1 s per well. The luminescence signal was recorded using a Synergy HT Microplate Reader (BioTek Instruments Inc., Winooski, VT, USA). All tests were performed in triplicates. Cells incubated without filaments were used as a positive control and cells incubated with 10%(*v/v*) dimethylsulfoxide (DMSO) solution as a negative control.

### 2.10. Magnetic Characterization in Static and Alternating Fields

The magnetic properties of the samples were characterized using a SQUID magnetometer MPMS 5S (LOT Quantum Design, San Diego, CA, USA). For the preparation of as synthesized MNP, 30 µL of the sample was mixed with 30 µL 15% (*w/w*) mannitol solution in a polycarbonate (PC) capsule and freeze dried at −85 °C and 0.04 mbar in a lyophilisator (Alpha 2-4 LDplus, Martin Christ Gefriertrocknungsanlagen GmbH, Osterode, Germany) for 12 h. The PP filaments were weighed and measured inside Teflon capsules placed in the sample holder. For the determination of the saturation magnetization values, magnetization measurements were performed at 295 K, varying the field strength from zero to 4·10^6^ A/m. From the fit with the Langevin function, the saturation magnetization *M*_S_ was determined (see Supplementary Materials S1). In order to determine the particle magnetic size *d*_m_, so-called Chantrell fitting according to literature was performed assuming log-normal distributed MNP sizes [65,66]. The ZFC magnetization curves were obtained by measuring the magnetization in a magnetic field of 796 A/m, while the temperature was stepwise increased from 5 to 295 K. The FC magnetization curves were obtained by magnetization measurements at the same field as for ZFC measurements, while the temperature was stepwise decreased from 295 to 5 K.

Magnetic particle spectroscopy (MPS) measurements were carried out using a commercial spectrometer (Bruker BioSpin MRI GmbH, Ettlingen, Germany) operated at *H* = 25 mT/μ_0_, *f* = 25 kHz, and 37 °C. A sample volume of 10 µL dispersed MNP as well as PP filaments were measured inside PCR tubes. From the measured amplitude spectrum (higher harmonics *A*_n_(*n f,*), *n* = 3, 5, 7, …), the concentration independent amplitude ratio *A*_5_/*A*_3_ was determined, which is an indicator of the state and environment of an MNP sample [67,68].

Alternating current susceptibility (ACS) was measured using a DynoMag susceptometer (RISE Acreo, Gothenburg, Sweden) covering a frequency range of 1 Hz to 500 kHz with a AC field amplitude of 360 A/m. The field amplitude of 360 A/m is stable up to a frequency of 15 kHz and then continuously decreases with increasing frequency to 20 A/m (at 500 kHz). Measurements were performed with 100 µL dispersed MNP at 295 K. The real *χ*’(*f*) and imaginary *χ*’’(*f*) magnetic susceptibility was acquired.

### 2.11. Characterization of Heating Efficiency

The inductive heating experiments were performed with a custom-build hyperthermia setup (Trumpf Hüttinger GmbH + Co. KG, Freiburg, Germany), which consists of a DC generator, an AC-resonant oscillator, and a water-cooled copper coil (inductor). For the experiments, an AMF was generated with frequencies of 95, 140, and 270 kHz, respectively, and field amplitudes ranging from 14 to 65 kA/m. The field parameters (frequency and field amplitude) were verified by measurements with a magnetic field probe (NanoScience Laboratories Ltd., Larchwood, UK). A sample volume of 1 mL was prepared in 4 mL glass vials, placed in the center of the inductor, and exposed to the AMF for 30 min starting at an initial temperature of *T*_0_ = 37 °C. In order to prevent vaporization of water, each glass vial was covered with parafilm during the measurement. The temperature was recorded with a fiber optical sensor (Luxtron 812, LumaSense Technologies Inc., Santa Clara, CA, USA). The dispersed MNP were diluted to an iron concentration of 1.1 mg(Fe)/mL, which corresponds to the iron concentration of the PP@3%MNP filaments. An amount of 50 mg (approximately 1 mm long) PP filaments was stacked in the form of a thin layer and placed inside 1 mL agarose hydrogel with a monomer mass fraction of 1.5%(*w/w*). A drawing and an exemplary photograph of a sample are available in Supplementary Materials S3 (Figure S3). Each hybrid stent was placed vertically in a plastic tube and subsequently embedded in 10 mL agarose hydrogel (approximately 1 mm of the stent edges protrude from the hydrogel). For the preparation of the agarose hydrogel, agarose was mixed with deionized water, heated up to 90 °C for 30 min and cooled down under ambient conditions after preparation. For background subtraction, reference measurements of water and agarose hydrogel were performed for each AMF setting. The measured temperature data, *T*(*t*), was fitted with the Box–Lucas function to determine the specific loss power value (SLP) [69] (see Supplementary Materials S1). The temperature profile during heat dissipation in the surroundings of the hybrid stents was continuously recorded using a thermographic camera (testo 882, Testo SE & Co.KG, Titisee-Neustadt, Germany) with an emission coefficient *α* = 0.96. The temperature difference was illustrated after background subtraction. The thermographic camera results were validated by simultaneous temperature measurement at the stent surface using a fiber-optic thermometer. For the investigation of heating efficiency, the ambient conditions were kept constant, and the field settings were controlled before each measurement. The measurements were performed following a standardized protocol. The uncertainty analysis includes the uncertainty of MNP concentration determination as well as the slight variations of the AMF parameters and of the ambient conditions [70].

### 2.12. Feasibility of Endoscopic Placement

A hybrid stent St@5%MNP was placed into the bile duct of a porcine cadaver after explantation of the bile duct and its surrounding tissues. The animal cadaver was taken from other approved animal experiments directly after euthanization. No additional ethical approval was needed according to German law and in agreement with the LANUV (State Agency for Nature, Environment, and Consumer Protection, North Rhine-Westphalia). All animal-handling procedures were performed in concordance with the Guide for the Care and Use of Laboratory Animals of the National Institutes of Health and followed the guidelines of the Animal Welfare Act. The hybrid stent St@5%MNP located in the bile duct and the surrounding tissues were then fixated in the described hyperthermia set-up. After hyperthermia treatment at 45 °C for 1 h, the bile duct tissue was fixed in formalin and cut into slices with a thickness of approximately 2–3 µm. The samples were dewaxed using alcohol-xylene series. For H&E staining, first undiluted Mayer’s hematoxylin (Merck, Darmstadt, Germany) and subsequently 1% eosin (Merck, Darmstadt, Germany) were used. The samples were washed in 0.4% acetic acid and covered with Vitro-Clud (Langenbrinck GmbH, Emmendingen, Germany).

## 3. Results and Discussion

**Figure 1:**
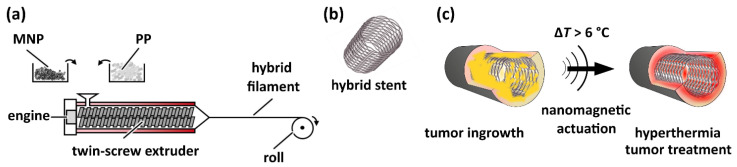
(**a**) Illustration of the production process of hybrid filaments via melt-spinning of PP pellets and MNP with a twin-screw extruder. (**b**) Sketch of a hybrid stent. (**c**) Illustration of hyperthermia approach to treat hollow organ tumors using a hybrid stent: The MNP inside the stent are actuated in an alternating magnetic field, leading to a temperature increase by more than 6 °C at the stent surface. This local overheating leads to the destruction of the tumor (colored in yellow) near the stent surface.

Figure 1 schematically depicts the production process of hybrid filaments via melt-spinning of polypropylene (PP) and MNP powder with a twin-screw extruder, the further assembly to stents, as well as their envisaged application for the treatment of hollow organ tumors. Three different hybrid filaments with a diameter of (450 ± 80) µm and with 3%(*w/w*), 5%(*w/w*), and 7%(*w/w*) MNP loading (denoted as PP@3%MNP, PP@5%MNP, and PP@7%MNP) were successfully produced. Using these filaments, three different stents were braided (denoted as St@3%MNP, St@5%MNP, and St@7%MNP).

### 3.1. Physico-Chemical Properties of MNP and Hybrid Filaments

The basic properties of the dispersed MNP sample as well as of the three hybrid filaments PP@3%MNP, PP@5%MNP, and PP@7%MNP incorporated with 2.7% (*w/w*), 4.4% (*w/w*), and 7.2% (*w/w*) MNP, respectively, are summarized in Table 1. Further details on the iron concentration inside the PP filaments determined by TGA (including the TGA curves) and PA measurements are presented in Supplementary Materials S2 Table S2, respectively, Figure S2.

**Figure 2:**
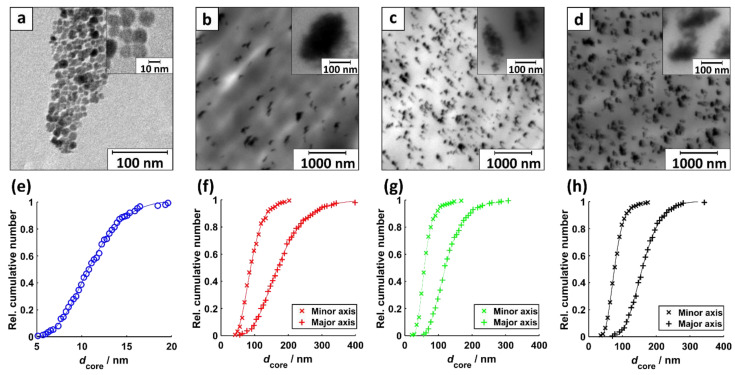
TEM images of (**a**) MNP disp, (**b**) MNP inside PP@3%MNP, (**c**) PP@5%MNP, and (**d**) PP@7%MNP filaments. The insets show an exemplary magnification of one part of the same image. The corresponding core diameter distribution with a fit to a log-normal CDF of (**e**) spherical shaped dispersed MNP (*R*^2^ = 0.9987), (**f**) minor and major axis of ellipsoidal shaped MNP agglomerated inside PP@3%MNP (*R*^2^_min_ = 0.9984, *R*^2^_maj_ = 0.9992), (**g**) PP@5%MNP (*R*^2^_min_ = 0.9991, *R*^2^_maj_ = 0.9991), and (**h**) PP@7%MNP filaments (*R*^2^_min_ = 0.9995, *R*^2^_maj_ = 0.9992).

Figure 2 displays exemplary transmission electron microscopy (TEM) images of dispersed MNP, MNP inside PP@3%MNP, PP@5%MNP, and PP@7%MNP filaments. The images show circular-shaped MNP (Figure 2a) and ellipse-shaped MNP agglomerates (Figure 2b–d).

**Figure 3:**
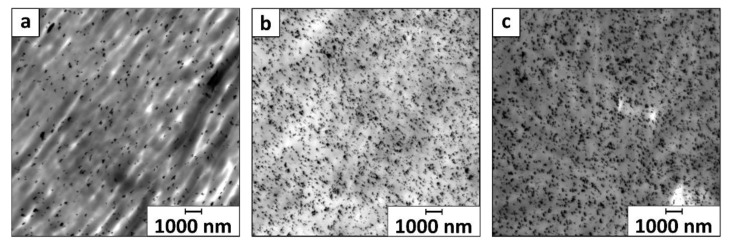
Overview TEM images of (**a**) MNP inside PP@3%MNP, (**b**) PP@5%MNP, and (**c**) PP@7%MNP filaments displayed with a magnification factor of 600. The displayed area is approximately 225 µm^2^.

The geometry of the MNP agglomerates arises presumably from axial shear stress during the extrusion process, in which the PP and MNP mixture is pressed through a nozzle. The TEM analysis yields log-normal distributed single and agglomerated MNP sizes (Figure 2e–h). For the ellipse-shaped agglomerated MNP, the mean size was analyzed for the major and minor axes (see Figure 2f–h). The results of the mean core size determination are summarized in Table 1. For all filament types, PP@3%MNP, PP@5%MNP, and PP@7%MNP, the ratio between minor and major axis of the MNP agglomerate size is about 1/2. The mean size of the agglomerates is more than ten times higher than the core size of the MNP. Increasing MNP concentration inside the filaments obviously does not lead to the formation of bigger agglomerates (see Table 1 and Figure 3). The largest relative difference in the agglomerate sizes is observed for PP@3%MNP compared to one of PP@5%MNP (37% for minor axis and 30% for the major axis).

Exemplary TEM images in Figure 3 display uniformly distributed small MNP agglomerates inside PP@3%MNP, PP@5%MNP, and PP@7%MNP filaments, which according to literature lead to better interfacial interactions and enhanced mechanical properties of the hybrid filaments [71].

**Figure 4:**
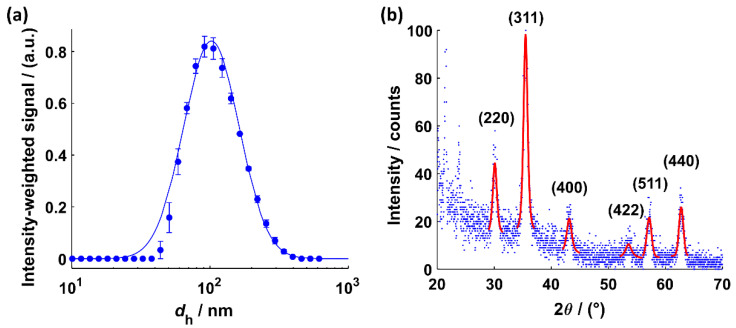
(**a**) Intensity-weighted size distribution of the hydrodynamic diameters with a fit (*R*^2^ = 0.9973) to the PDF of a log-normal distribution for the MNP dispersed in water. (**b**) XRD intensity profile for MNP dispersed in water with a fit of the Pseudo-Voigt function to determine the corresponding Bragg angles (see Supplementary Materials S4 for *R*^2^ values).

**Figure 5:**
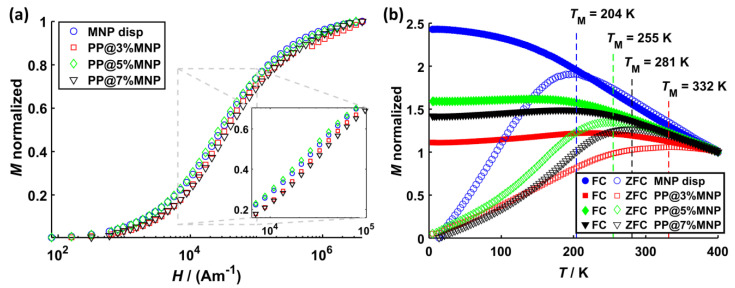
(**a**) Virgin magnetization curves as well as (**b**) ZFC (open symbols) and FC (filled symbols) magnetization curves for dispersed MNP, PP@3%MNP, PP@5%MNP, and PP@7%MNP. The virgin curves are normalized to the saturation magnetization, while ZFC/FC curves are normalized to the initial magnetization value of the FC curve.

Figure 4a shows the result of the DLS measurement for MNP dispersed in water. The PDF fit to the intensity-weighted size distribution of the dispersed MNP yields an MNP hydrodynamic diameter of *d*_h_ = (141 ± 69) nm. The XRD data and Bragg angles are shown in Figure 5b. The intensity profile between 20° and 30° can be attributed to crystalline lauric acid [72]. A clear delineation between a Fe_3_O_4_ and a γ-Fe_2_O_3_ crystal structure according to the determined Bragg angles is not feasible. MNP are known to show significant peak broadening and poor statistics in the XRD pattern, which makes it difficult to detect the very small differences in the diffraction intensity patterns of Fe_3_O_4_ and γ-Fe_2_O_3_. An investigation of the crystal structure of MNP inside the polymer matrix by XRD measurements is not possible. For that, extensive electron energy loss measurements are necessary [18]. However, the Bragg angles and their corresponding FWHM values were used to determine a weighted average of the crystal size *d*_cryst_ = (9.5 ± 0.4) nm (see Supplementary Materials S4 Table S4). The result is in line with the findings from TEM measurements (*d*_cryst_ = (9.5 ± 0.4) nm, see also Table 1).

The normalized magnetization curves for all samples are shown in Figure 5 and the hysteresis curves are shown in Supplementary Materials S5 (Figure S5). The saturation magnetization *M*_S_ and the magnetic diameter *d*_m_ deduced from these curves as described in Section 2 Materials and Methods are listed in Table 1. From the magnetization curves, it can be deduced that stronger MNP dipole–dipole interactions arise because of agglomerate formation inside the filaments. This is indicated by the slightly lower magnetization values (Figure 5a) as well as by the broader shifted ZFC peaks towards higher temperatures for all PP filaments compared to the one of dispersed MNP. Such effects were associated with dipole–dipole interactions inside nanoclusters before [73,74,75]. Further, the shift and broadening of the ZFC peak relates to the size of MNP agglomerates and is, for this case, most pronounced for PP@3%MNP.

The effective anisotropy constant *K*_eff_ is estimated from the peak temperatures *T*_M_ of the ZCF curve and the magnetic volume $V_{m}{=(\pi /6)d}_{m}^{3}$ using the relation $K_{\mathrm{eff}}V_{m}\approx 23.6\;k_{B}T_{M}$ [76] and yields $K_{\mathrm{eff}}\;\approx {\;120\;\mathrm{kJ}/m}^{3}$ (MNP disp), $K_{\mathrm{eff}}\;\approx {\;226\;\mathrm{kJ}/m}^{3}$ (PP@3%MNP), $K_{\mathrm{eff}}\;\approx {\;123\;\mathrm{kJ}/m}^{3}$ (PP@5%MNP), and $K_{\mathrm{eff}}\;\approx {\;151\;\mathrm{kJ}/m}^{3}$ (PP@7%MNP). This is a rough estimation of *K*_eff_, which does not take into account effects arising from MNP size distribution or surface anisotropy; however, it qualitatively demonstrates an anisotropy increase with larger MNP agglomeration size, which is in line with the findings in literature [77,78]. The high anisotropy values are explained by MNP dipole–dipole interactions inside MNP agglomerates as discussed above. $K_{\mathrm{eff}}$ values from literature of non-interacting magnetite nanoparticles with similar core sizes are in the range of 23 to 41 kJ/m³ [79].

**Figure 6:**
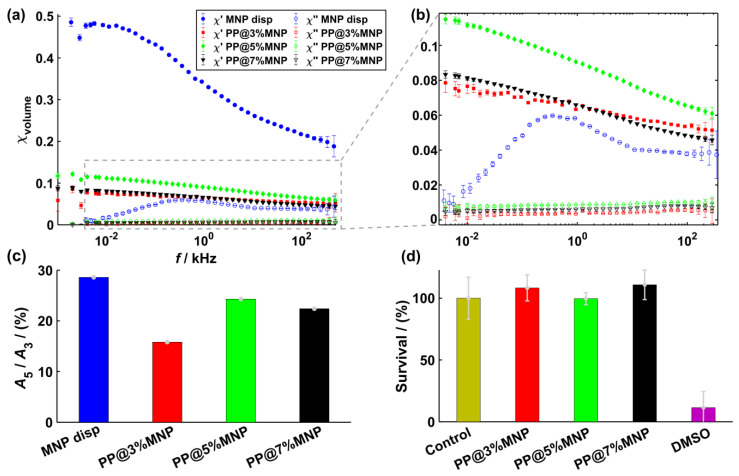
(**a**) Real part χ′ (filled symbols) and imaginary part χ″ (open symbols) of the complex dynamic susceptibility for dispersed MNP, MNP inside PP@3%MNP, PP@5%MNP, and PP@7%MNP filaments. (**b**) Zoom of the volume susceptibility at lower values. (**c**) *A*_5_/*A*_3_ amplitude ratios for dispersed MNP, MNP inside PP@3%MNP, PP@5%MNP, and PP@7%MNP filaments. (**d**) Survival analysis of L929 cells after 24 h incubation with hybrid filaments (PP@3%MNP, PP@5%MNP, and PP@7%MNP).

The results from AC-susceptibility measurements are shown in Figure 6a and display the real ($\chi^{\prime }$) and the imaginary ($\chi^{\prime \prime }$) part of the volume susceptibility for each sample. Figure 6b shows the same results at smaller susceptibility values. $\chi^{\prime \prime }$ values of MNP inside PP@3%MNP, PP@5%MNP, and PP@7%MNP filaments are smaller compared to the ones of dispersed MNP. Only for dispersed MNP, a broad $\chi^{\prime \prime }$ peak occurs at approximately 3 kHz. Because of the absence of an $\chi^{\prime \prime }$ peak for MNP immobilized inside the hybrid filaments and with the assumption of magnetically blocked MNP at 3 kHz, the peak can be related to Brownian relaxation effects [80]. Such a decrease of the ACS signal was previously associated with the immobilization of MNP inside hydrogels [45]. In this case, the relaxation of the MNP is dominated by the Néel relaxation and dipole–dipole interactions strongly influence the MNP relaxation dynamics. Consequently, the classical Debye model of the classical Linear Response Theory [79] used for the description of MNP relaxation dynamics is no longer valid. An extension of this model by considering a distribution of relaxation times, the so-called Cole–Cole model, was proposed to describe the relaxation phenomena of MNP inside magnetic scaffolds [41,43]. The ACS results in Figure 6 qualitatively describe the MNP relaxation behavior according to the Cole–Cole model. In the frequency range used for hyperthermia experiments (approximately 100 kHz and higher), the relative difference between $\chi^{\prime \prime }$ of MNP inside filaments and dispersed MNP is approximately 85%. Moreover, $\chi^{\prime }$ values of PP@5%MNP are higher than for PP@3%MNP and PP@7%MNP, which seems to relate to the MNP agglomerate size. Further, the smooth decay of $\chi^{\prime }$ values for PP@3%MNP indicates stronger dipole–dipole interactions [81].

Figure 6c shows the amplitude ratios *A*_5_/*A*_3_ obtained by MPS for the dispersed MNP and MNP inside the PP filaments, respectively. *A*_5_/*A*_3_ decreases by approximately 45% for PP@3%MNP compared to dispersed MNP. For PP@5%MNP and PP@7%MNP, a less pronounced decrease of only 15% and 22% is observed. Such decrease in *A*_5_/*A*_3_ was recently reported for immobilized particles [37] and for particles agglomerated inside living cells [82]. Furthermore, this trend confirms the presence of stronger dipole–dipole interactions inside PP@3%MNP filaments as already deduced from the ZFC peak shift (Figure 5b) and susceptibility values (Figure 6a).

Figure 6d shows the effects on the cell survival after 24 h incubation with PP@3%MNP, PP@5%MNP, and PP@7%MNP filaments, respectively. The filaments showed no cytotoxic effect.

### 3.2. Heating Efficiency

MNP agglomeration and immobilization obviously influences the static and dynamic magnetization behavior of the hybrid filaments. The dipole–dipole interaction and the effective anisotropy energy increase directly influences the non-linear dynamic magnetic susceptibility of the MNP [83]. Since MFH relies on the non-linear dynamic magnetic susceptibility, a significant influence on the heating efficiency is expected. To investigate this, the SLP values of MNP dispersed in water, MNP immobilized in acrylamide hydrogel, PP@3%MNP, PP@5%MNP, and PP@7%MNP are determined. The investigation of MNP immobilized in hydrogels aims at the quantification of the immobilization effect neglecting agglomerations.

**Figure 7:**
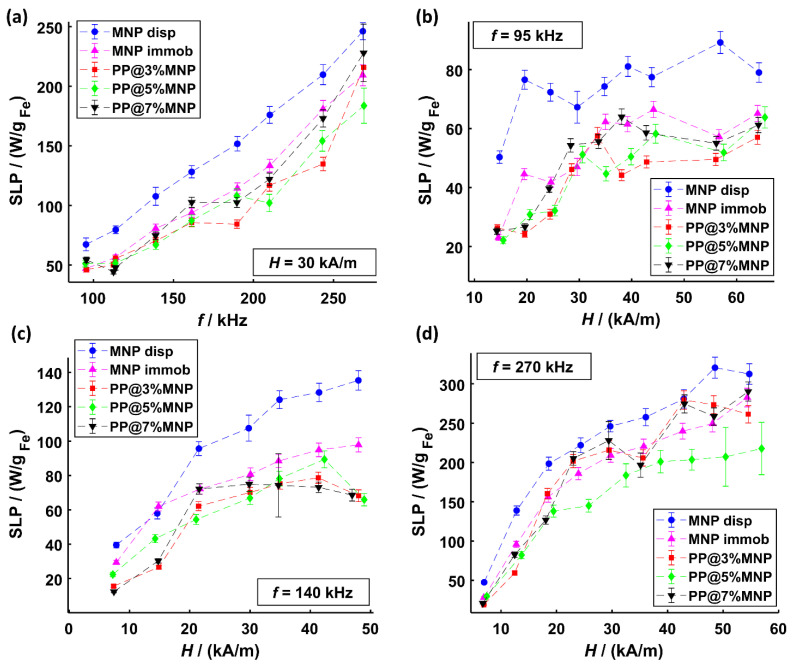
SLP values of MNP dispersed in water and MNP immobilized inside hydrogels as well as inside PP@3%MNP, PP@5%MNP, and PP@7%MNP filaments for (**a**) different frequencies at a constant magnetic field amplitude of 30 kA/m, (**b**) different magnetic field amplitudes at 95 kHz, (**c**) different magnetic field amplitudes at 140 kHz, and (**d**) different magnetic field amplitudes at 270 kHz.

In Figure 7a, the SLP results at a magnetic field amplitude of 30 kA/m with gradual frequency increase from 95 to 270 kHz are shown. The values range from approximately 50 up to 240 W/g_Fe_ and indicate a linear increase with frequency. The SLP dependence of magnetic field amplitude (form approximately 14 to 65 kA/m) for three different frequencies (95, 140, and 270 kHz) is shown in Figure 7b–d. Overall, the SLP values increase with field amplitude until saturation is reached. The highest SLP value of approximately 300 W/g_Fe_ is reached at the highest frequency *f* = 270 kHz and field amplitude *H* = 55 kA/m. In some cases, an “s-shaped” increase of SLP values is observed. This kind of SLP relation to magnetic field amplitude was reported in literature before for magnetosomes immobilized in agarose [84] and explained by the chain structure of magnetosomes accelerating magnetic relaxation. Similarly, certain structures of MNP agglomerates inside the filaments could influence the time-dependent relaxation processes in a magnetic field. SLP saturation effects, however, showing a steep increase with magnetic field amplitude are well known and were reported in literature for different dispersed nanoparticle samples at frequencies ranging from 150 up to 375 kHz [85,86,87].

In Figure 7, a clear difference in heating efficiency between immobilized MNP and MNP dispersed in water can be observed. This difference is attributed to the blocked Brownian relaxation of MNP. With increasing magnetic field amplitude, the relative SLP difference between dispersed MNP and immobilized MNP (in hydrogel and filaments, respectively) decreases until a certain saturation is reached (at about 30 kA/m). Such SLP decrease by up to 35% at 270 kHz was reported in literature before for 9 nm iron oxide MNP immobilized in hydrogels [39]. The lowest relative heating efficiency differences between dispersed and immobilized MNP are observed at 270 kHz, indicating that at higher frequencies, Néel relaxation dominates. The SLP values of MNP inside hybrid filaments are in most cases lower than those immobilized in hydrogel. These differences can be explained by MNP agglomeration diminishing the heating efficiency and are in agreement with findings from literature [88]. Various sizes of MNP agglomerates may also explain the different SLP values reached for different hybrid filaments. In such cases, the change in the effective anisotropy energy due to dipole–dipole interactions, which is dependent on the MNP agglomerate size (see also magnetization measurements in Figure 5), must be regarded. Monte-Carlo simulations suggest that dipole–dipole interactions lead to significant and complex effects on the energy dissipation rate and that the effects are dependent on intrinsic statistical properties of the MNPs [89].

Such agglomeration and immobilization effects were also observed in the measurements of the imaginary part of the complex dynamic susceptibility $\chi^{\prime \prime }$ (see Figure 6a,b) as well as *A*_5_/*A*_3_ ratio (see Figure 6c) for the same samples. Generally, for MNP agglomerates, the interparticle interactions play an important role leading to either an increase or decrease of SLP depending on MNP’s magnetocrystalline properties and the structure of agglomerates. MNP chain formation is associated with an amplified heating efficiency due to long range collective magnetic behavior reducing the magnetostatic energy [84,90]. In contrast, for large and dense centrosymmetric agglomerates, a reduced SLP is often observed and attributed to demagnetization effects, causing a weakened coupling to the external AMF [88,91]. Magnetic moments of MNP inside agglomerates especially at low magnetic field amplitudes increasingly fail to overcome the dipolar field [92]. As recently reported, small Néel dominated MNP immobilized in filaments show especially at small field amplitudes higher heating efficiencies compared to large ferromagnetic MNP clusters of high anisotropy [93]. To overcome the agglomeration driven SLP diminution, other polymers with lower melting temperature, e.g., low-density polyethylene (LDPE), in combination with a temperature resistant particle coating (e.g., silica) can be used to achieve the distribution of individual particles inside the filaments.

### 3.3. Nanomagnetic Actuation of Hybrid Stents

**Figure 8:**
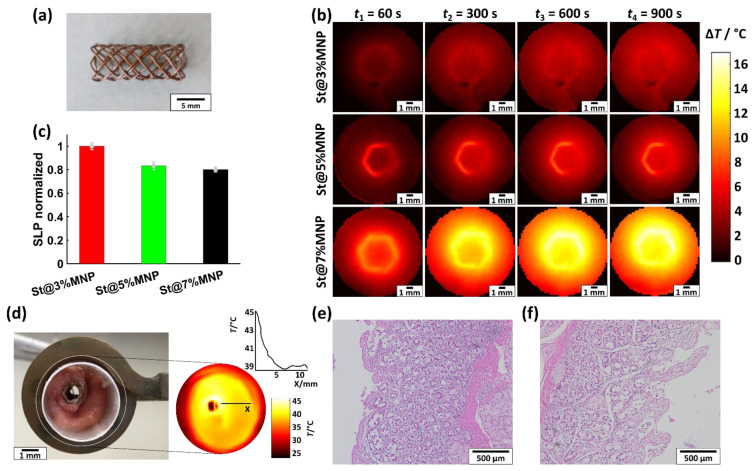
(**a**) Photograph of an exemplary St@7%MNP stent. (**b**) Temperature rise Δ*T* of St@3%MNP, St@5%MNP, and St@7%MNP during the application of an AMF with *f* = 270 kHz and *H* = 54 kA/m with a thermographic camera at *t*_1_ = 60 s, *t*_2_ = 300 s, *t*_3_ = 600 s, and *t*_4_ = 900 s. (**c**) Normalized SLP values of St@3%MNP, St@5%MNP, and St@7%MNP (absolute SLP values are provided in Table S6 in Supplemantary Materials S6). (**d**) Photograph of an exemplary St@5%MNP stent inserted into a resected bile duct of a pig. The inset shows the temperature distribution during the application of an AFM with *f* = 270 kHz and *H* = 38 kA/m. (**e**) HE staining images of the heated bile duct and (**f**) an untreated reference bile duct.

For hollow organ tumor treatment, the hybrid stents must be able to generate uniformly distributed heat with temperatures of approximately 43 °C in the surrounding tissue. The feasibility of meeting these demands is demonstrated for three prototype bile duct stents St@3%MNP, St@5%MNP, and St@7%MNP (each 5 mm × 16 mm), which are made using PP@3%MNP, PP@5%MNP, and PP@7%MNP filaments, respectively. For this, the temperature rise Δ*T* of the stents (at *f* = 270 kHz and *H* = 54 kA/m) is measured with a thermographic camera placed above the opening of the stents and with a fiber-optic thermometer placed in close vicinity of the stent surface (Figure 8).

Figure 8a shows a picture of an exemplary stent. In Figure 8b, the temperature rise Δ*T* in the surrounding hydrogel at selected AMF exposure times *t*_1_ = 60 s, *t*_2_ = 300 s, *t*_3_ = 600 s, and *t*_4_ = 900 s is displayed. At 900 s, saturation is reached. The entire heating process up to *t*_max_ = 1000 s was recorded using a thermographic camera and is provided as a video in the Supplementary Materials. Δ*T* values of St@3%MNP, St@5%MNP, and St@7%MNP from the thermographic measurements reach values up to 6, 9, and 17 °C, respectively. Based on a body temperature of 37 °C, this corresponds to a heating of the tissue of 43, 46, and 54 °C. Heat uniformly dissipated inside the hydrogel surrounding the stents until an equilibrium was achieved. For all stents, a temperature rise inside the hydrogel up to a distance of approximately 8 mm to stent surface could be measured. The absolute temperature values strongly depend on the amount of MNP inside the filaments.

To compare the heating efficiency of the stents, their SLP values were calculated using the Δ*T* values after 1000 s. Δ*T* was measured with a fiber optical sensor placed in close vicinity to the stent surface. Figure 8c shows the normalized SLP values resulting from these measurements. Compared to St@3%MNP, St@5%MNP and St@7%MNP have a 16% and 20% lower heating efficiency, respectively.

For a first feasibility test of endoscopic stent placement, an exemplary hybrid stent St@5%MNP was crimped and inserted into the explanted bile duct of a pig. By setting the AMF to *f* = 270 kHz and *H* = 38 kA/m, the stent elevated the tissue temperature to 45 °C at the stent surface for one hour. The thermographic image in Figure 8d shows the temperature distribution in the surrounding tissue. The HE staining images of the bile duct tissue after the hyperthermia treatment compared to healthy bile duct tissue (Figure 8e,f) revealed no difference in histology, especially no signs of direct tissue damage.

Nanoactuation of hybrid stents is a promising nanotechnology that enables local hyperthermia treatment with significant advantages over conventional magntic fluid hyperthermia approaches, such as high local MNP concentration. Furthermore, it is easily predictable and allows controllable heat release. The results demonstrate the feasibility of nanomagnetic actuation of three prototype hybrid stents for hyperthermia treatment of hollow organ tumors. Depending on MNP load and field settings, a temperature rise by up to 17 °C could be achieved. In literature, various temperature rises were reported, such as 20 °C for gelatin-based scaffolds, 13 °C for ceramics, or 9 °C for PCL-based materials [28,30]. As previously reported for magnetic scaffolds, the heating perfomence of the hybrid stents is strongly influenced by the change in magnetic relaxation dynamics due to interparticle interactions and blocking of the Brownian relaxation. To describe the relaxation dynamics of MNP in magnetic scaffolds, the Cole–Cole model was proposed [41,42,43]. Moreover, for a deeper understanding of the fundamentals of the magnetic relaxation processes of MNP and prediction of the heating efficiency of MNP inside hybrid materials, non-equilibrium stochastic simulations of the relaxtions dymanics proved to be a powerful tool [46,47]. For clinical applications, safe AMF exposure of the patient must be adhered. The safety limit is defined by the product between field amplitude and frequency (*H*·*f*)_crit_, which must not exceed a certain critical value. However, several critical values are still under discussion. For clinically available hyperthermia devices, a safety limit of (*H*·*f*)_crit_ = 1.8 × 10^9^ A·m^−1^s^−1^ is considered [32]. FDA guidelines for MRI imaging assume safe values as (*H*·*f*)_crit_ = 3.8 × 10^9^ A·m^−1^s^−1^ [94]. For small body regions and considering the severity of the disease, a higher safety limit of (*H*·*f*)_crit_ = 5 × 10^9^ A·m^−1^s^−1^ is suggested [95]. Even higher values of (*H*·*f*)_crit_ = 6.7 × 10^9^ A·m^−1^s^−1^ were tested and well tolerated in in vivo experiments on mice [96]. For the hybrid stents in this work, the optimization of AMF settings under considerations of safety limits in in vivo trials is envisaged. However, there is an onging discussion about the critical values defining the safety limits. Regulations and standardized protocols for hyperthermia applications have still to be established [97,98]. Guidelines for interstitial hyperthermia suggest a target temperature range of 40 to 44 °C for a period of 30 to 60 min depending on the tumor type. For the clinical translation of the therapeutical approach with nanoactuation of hybrid stents, quality assurance quidelines such as the ones developed for interstitial hyperthermia [99] are necessary. Detailed instructions on characterization and documentation of the performance of hybrid stent induced hyperthermia to achieve reproducible results must be provided. This includes workflow and procedures as well as technical requirements. For this, the development of specific guidelines for intraluminal hyperthermia is recommended.

## 4. Conclusions

In this paper, we describe a magnetic nanotechnology that locally activates hyperthermia treatment of hollow organ tumors. This is enabled by using a polymer hybrid stent with incorporated magnetic nanoparticles (MNP), which is activated in an alternating magnetic field (AMF) to release therapeutically effective heat in a controlled manner. Our investigations demonstrate the feasibility of such nanotechnology for three prototype bile duct hybrid stents with three different MNP concentrations (3%(*w/w*), 5%(*w/w*), and 7%(*w/w*)). The stents showed uniformly distributed heat on their surface and inside the surrounding hydrogel as well as inside explanted bile duct of a pig. The parameters controlling heating performance of the hybrid stents such as AMF parameters, MNP immobilization, and agglomeration were thoroughly investigated with different techniques to quantify their impact on heating efficiency. Independent of MNP concentration, the heating efficiency of stent filaments showed an increase with frequency from approximately 60 W/g_Fe_ at 95 kHz to approximately 250 W/g_Fe_ at 270 kHz. Further, gradual increase of the magnetic field amplitude resulted in a steep increase in heating efficiency until saturation at approximately 30 kA/m was reached. The effect of MNP immobilization on heating efficiency was more pronounced at lower frequencies and magnetic field amplitudes, showing a relative difference in heating efficiency of up to 60% compared to that of freely dispersed MNP. This difference was attributed to the absence of Brownian relaxation to the overall magnetic relaxation process. At higher frequencies and magnetic field amplitudes, such relative difference significantly decreased indicating the domination of Néel relaxation. In most cases, MNP agglomeration showed only a slight decrease in heating efficiency, which was attributed to interparticle interactions. These findings were validated with measurements in static magnetic field (SQUID magnetometry) and dynamic magnetic fields (MPS and AC susceptibility). In conclusion, generation of therapeutically effective temperatures of 43 °C at the surface of prototype stents, e.g., for the bile duct, with uniformly distributed heat in its surrounding is feasible. This was accomplished by adjusting AMF parameter with regard to MNP properties to control the heating performance. Similar to the results presented in literature for magnetic scaffolds, MNP immobilization and agglomeration effects play an important role in the hyperthermia performance of hybrid stents. Due to the change in MNP effective anisotropy and blocking of Brownian relaxation directly influencing the MNP magnetic relaxation dynamics, the heating efficiency of MNP in the hybrid stents is lower than that of freely dispersed MNP. To enhance the heating efficiency, AFM settings must be adjusted, e.g., higher amplitudes have to be applied. Considering medical safety limits of electromagnetic exposure of the patient, further investigations are necessary to find the best match between AFM settings and MNP properties, to reach the necessary therapeutically effective heat for the lowest possible AFM exposure. As controlling heat release is a complex interplay between field and MNP parameters, models taking into account interparticle interactions, dynamic magnetic relaxation, and anisotropy energy changes of the MNP are necessary for the accurate prediction of the heat performance of hybrid stents. First investigations demonstrated the biological safety of the hybrid stents. The investigation of therapeutical effects of the hybrid stent hyperthermia treatment are envisaged.

## Figures and Tables

**Table 1:** Physico-chemical properties of MNP dispersed in water (MNP disp) and hybrid filaments (PP@3%MNP, PP@5%MNP, and PP@7%MNP): iron concentration *c*_Fe_, core diameter *d*_core_ (for the filaments the major and minor axis of agglomerates is specified), hydrodynamic diameter *d*_h_, crystal diameter *d*_cryst_, magnetic diameter *d*_m_, saturation magnetization *M*_S_, and maximum temperature *T*_M_ in the ZFC curve. *σ* denotes the standard deviation.

Property	MNP Disp	PP@3%MNP	PP@5%MNP	PP@7%MNP
(*c*_Fe_ ± σ_core_)/%(*w/w*)	-	1.97 ± 0.11	3.20 ± 0.11	5.24 ± 0.11
(*d*_core_ ± σ_core_)/nm	11.3 ± 3.3	Maj. Ax.: 181 ± 71 Min. Ax.: 93 ± 32	Maj. Ax.: 127 ± 46 Min. Ax.: 60 ± 20	Maj. Ax.: 164 ± 46 Min. Ax.: 79 ± 23
(*d*_h_ ± σ_h_)/nm	141 ± 69	-	-	-
(*d*_cryst_ ± σ_cryst_)/nm	9.5 ± 0.4	-	-	-
(*d*_m_ ± σ_m_)/nm	10.2 ± 3.1	9.7 ± 2.4	10.9 ± 2.9	10.5 ± 2.9
(*M*_S_ ± σ_M_)/(Am^2^/kg(Fe))	99.4 ± 0.8	111 ± 6	98 ± 3	97 ± 2
*T*_M_/K	~204	~332	~255	~281

## Data Availability

The data that support the findings of this study are available from the corresponding author, I.S., upon reasonable request.

## Supplementary Materials

The following Supplementary Materials are available online at https://www.mdpi.com/2079-4991/11/3/618/s1: Table S2, Figure S2, Figure S3, Table S4, Figure S5, Table S6 and thermal videos of the entire heating process of stents: “Temperature_distribution_St@3%MNP”, “Temperature_distribution_St@5%MNP” and “Temperature_distribution_St@7%MNP”.
