# Supplementary material for: Nanomagnetic Actuation of Hybrid Stents for Hyperthermia Treatment of Hollow Organ Tumors
Source: Nanomaterials (Basel). 2021 Mar 2;11(3):618. doi: 10.3390/nano11030618 (PMC7999083; doi:10.3390/nano11030618)
Supplement: Supplementary file 1 [file nanomaterials-11-00618-s001.zip › Supplementary Materials/Supplementary Materials.pdf]

Supplementary Materials

Nanomagnetic Actuation of Hybrid Stents for Hyperthermia Treatment of Hollow Organ Tumors

Benedikt Mues, [a] Benedict Bauer, [b] Anjali A. Roeth [c], Jeanette Ortega, [b] Eva M. Buhl, [d] Patricia Radon, [e] Frank Wiekhorst, [e] Thomas Gries, [b] Thomas Schmitz-Rode [a] and Ioana Slabu*[a]

[a]Institute of Applied Medical Engineering, Helmholtz Institute, Medical Faculty, RWTH Aachen University, Pauwelsstraße 20, 52074 Aachen, Germany

[b]Institut für Textiltechnik, RWTH Aachen University, Otto-Blumenthal-Straße 1, 52074 Aachen, Germany

[c]Department of General, Visceral and Transplant Surgery, RWTH Aachen University Hospital, Pauwelsstraße 30, 52074 Aachen, Germany

[d]Institute of Pathology, Electron Microscopy Facility, RWTH University Hospital Aachen, Pauwelsstraße 30, 52074 Aachen, Germany

[e]Physikalisch-Technische Bundesanstalt, Abbestraße 2-12, 10587 Berlin, Germany

Supplementary Materials S1

Transmission electron microscopy (TEM)

For the spherical shaped MNP, the core diameter d of about 150 particles was measured using the software GIMP and fitted with the cumulative log-normal distribution probability density function (CDF)

$$\mathrm{CDF}(d,\mu,\sigma) = \frac{1}{2}+\left(1+\mathrm{erf}\left(\frac{\ln(d)-\mu}{\sqrt{2\sigma}}\right)\right), \tag{1}$$

with the parameters, μ and σ, from which the mean and variance are calculated with $d_{\mathrm{core}} = \exp(\mu+\sigma^2/2)$ and $\sigma_{d_{\mathrm{core}}}^2 = \exp(2\mu+\sigma^2)\cdot(\exp(\sigma^2)-1)$, respectively. Here, $\mathrm{erf}(x) = 2/\sqrt{\pi}\cdot\int_0^x \exp(-t^2)dt$ denotes the error function. The fits were performed with MATLAB® using the "nonlinear least squares" fitting algorithm.

Hydrodynamic light scattering (DLS)

The mean $d_{\mathrm{h}} = \exp(\mu+\sigma^2/2)$ and variance $\sigma_{d_{\mathrm{h}}}^2 = \exp(2\mu+\sigma^2)\cdot(\exp(\sigma^2)-1)$ of the hydrodynamic size are obtained by fitting the log-normal distribution probability density function (PDF) to the measured intensity data:

$$\mathrm{PDF}(d,\mu,\sigma) = \frac{1}{\sqrt{2\pi}\cdot d\cdot\sigma}\cdot\exp\left(-\frac{(ln(d)-\mu)^2}{2\sigma^2}\right), \tag{2}$$

with the parameters, μ and σ. The fits were performed with MATLAB ® using the "nonlinear least squares" fitting algorithm.

X-ray diffraction (XRD)

The Bragg angles were determined using the pseudo-Voigt function as a fitting model:

$$V(2\vartheta) = A(\eta L(2\vartheta) + (1-\eta)G(2\vartheta)\ . \tag{3}$$

With $L(2\vartheta) = (1+((2\vartheta-x_0)/w)^2)^{-1}$ the Lorentz-function and $G(2\vartheta) = \exp(-\ln 2((2\vartheta - x_0)/w)^2)$ a Gaussian function. with the central peak position $x_0 = 2\vartheta_{\mathrm{Bragg}}$ and the full-width-half-maximum FWHM $= 2w$. Here, $\vartheta_{\mathrm{Bragg}}$ denotes the Bragg-angle and $\eta \in [0,1]$ the mixing parameter describing the fractions of Lorentz and Gaussian contribution. The fits were performed with MATLAB® using the "nonlinear least squares" fitting algorithm. The MNP crystalline size d_{cryst} was determined using the modified Scherrer equation, $d_{\mathrm{cryst}} = K\cdot\lambda_{\mathrm{K\alpha}}/(\mathrm{FWHM}\cdot\cos\vartheta_{\mathrm{Bragg}})$, with $K = 0.9$ the shape factor (for spherical objects).

SQUID magnetometry

The Langevin function is defined as

$$L(\xi) = \coth(\xi) - \frac{1}{\xi}, \tag{4}$$

with $\xi = \mu H/(k_{\mathrm{B}}T)$ where $\mu = V_{\mathrm{M}}M_{\mathrm{S}}$ denotes the particle magnetic moment. Here, V_{M} the mean magnetic volume, H the applied magnetic field and $k_{\mathrm{B}} = 1.38\cdot10^{-23}$ J/K the Boltzmann constant. The fit to the Langevin function was performed with MATLAB® using the "nonlinear least squares" fitting algorithm.

Characterization of heating efficiency

The measured temperature data, $T(t)$ was fitted with the Box-Lucas function:

$$T(t) = \Delta T_{\mathrm{max}}(1-\exp(-bt)) + T_0. \tag{5}$$

The fits were performed with MATLAB® using the "nonlinear least squares" fitting algorithm.

The fitting parameters were used to determine the specific loss power value (SLP) according to:

$$\mathrm{SLP} = \frac{c}{\rho}\frac{\mathrm{d}T}{\mathrm{d}t}\Big|_{t\to 0} = \frac{c}{\rho}\Delta T_{\max}\, b, \tag{6}$$

where c = 4.187 J(gK)$^{-1}$ denotes the specific heat capacity of water, ρ the fraction of the iron mass m(Fe) in relation of the total mass of the sample.

Supplementary Materials S2

The iron concentration of dispersed MNP and of MNP inside PP filaments was examined by thermogravimetric analysis (TGA) and by photometric absorption (PA) measurements (see manuscript). Both, TGA and PA measurements were performed in triplicates. In Table S1 the iron concentration $\boldsymbol{c}_{\mathbf{Fe}}$ and MNP concentration $\boldsymbol{c}_{\mathbf{MNP}}$ for **PP@3%MNP**, **PP@5%MNP**, and **PP@7%MNP** are listed. The number in the sample denotation (e. g. 7 in PP@7%MNP) implies the MNP concentration inside the hybrid filaments. In the article, this denotation was chosen for practical reasons to unambiguously differentiate between the investigated filaments. Using the results of TGA and PA measurements, a weighted mean was calculated.

Table S2: Iron concentration $\boldsymbol{c}_{\mathbf{Fe}}$ and MNP concentration $\boldsymbol{c}_{\mathbf{MNP}}$ of the hybrid PP filaments measured by thermogravimetric analysis (TGA) and photometric absorption (PA). The weighed mean was calculated by using the results from TGA and PA.

**Method**		**PP@3%MNP**	**PP@5%MNP**	**PP@7%MNP**
**TGA**	c_{Fe} / % (w/w)	1.95 ± 0.11	3.22 ± 0.11	5.27 ± 0.11
	c_{MNP} / % (w/w)	2.69 ± 0.15	4.45 ± 0.15	7.28 ± 0.15
**PA**	c_{Fe} / % (w/w)	2.15 ± 0.36	2.92 ± 0.43	4.34 ± 0.59
	c_{MNP} / % (w/w)	2.97 ± 0.50	4.03 ± 0.59	5.99 ± 0.82
**Weighted mean**	c_{Fe} / % (w/w)	1.97 ± 0.11	3.20 ± 0.11	5.24 ± 0.11
	c_{MNP} / % (w/w)	2.72 ± 0.15	4.42 ± 0.15	7.23 ± 0.15

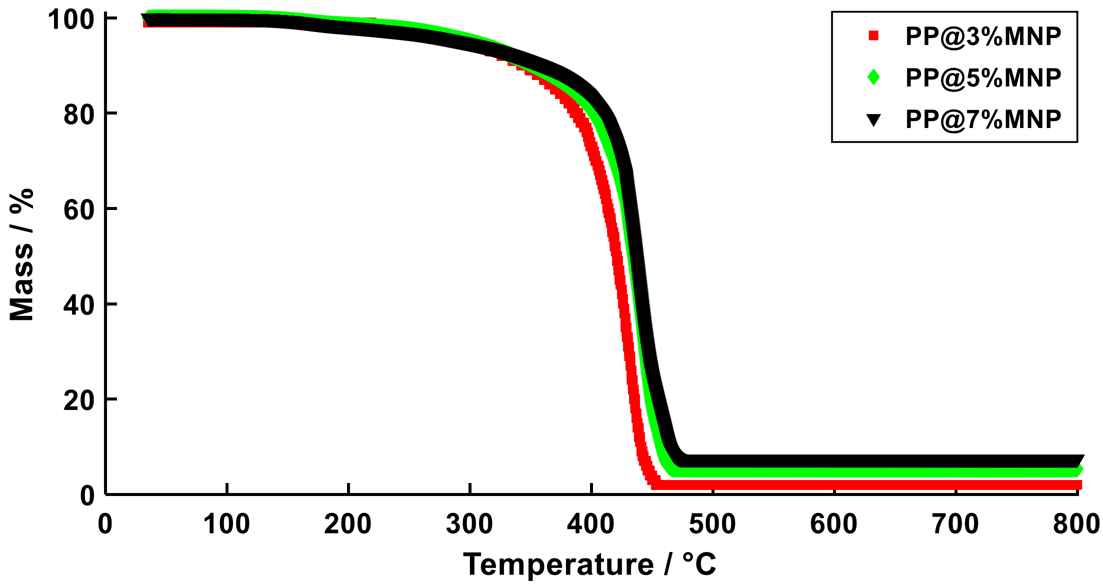

Figure. S2: TGA curves showing sample mass versus temperature for PP@3%MNP, PP@5%MNP and PP@7%MNP.

Supplementary Materials S3

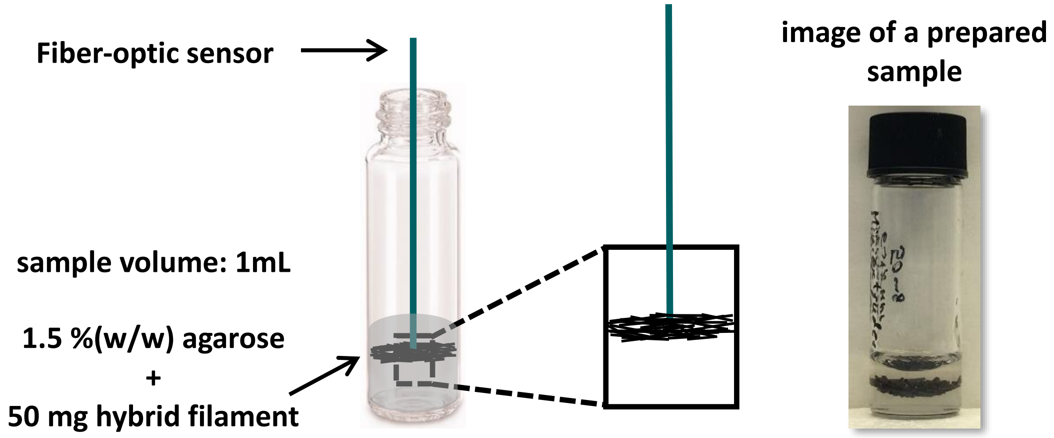

Figure. S3: Sketch and photo of a samples prepared for the hyperthermia measurement: 50 mg PP filaments (approximately 1 mm long) were stacked in the form of a thin layer and placed inside 1 mL agarose hydrogel.

Supplementary Materials S4

Table S.3 shows the Bragg angles $\boldsymbol{\vartheta}_{\mathbf{Bragg}}$ for each peak of the XRD intensity profile determined by fitting a Pseudo-Voigt function (see equation (3)). Furthermore, the reference Bragg angles $\boldsymbol{\vartheta}_{\mathbf{Bragg,Ref}}$ and corresponding reflections for magnetite from the International Center for Diffraction Data (ICDD) with reference PDF#85-1436 are listed. Using the modified Scherrer equation (see Supplementary Materials S1) the MNP crystalline diameter $\boldsymbol{d}_{\mathbf{cryst}}$ was calculated for each peak position (Table S3) and combined to d_{cryst} = (9.5 ± 0.4) nm as weighted average.

Table S4: Bragg angles $\boldsymbol{\vartheta}_{\mathbf{Bragg}}$ for each identified peak position and reference Bragg angles $\boldsymbol{\vartheta}_{\mathbf{Bragg,Ref}}$ with corresponding reflections from reference data for magnetite. Further, the MNP crystalline diameter $\boldsymbol{d}_{\mathbf{cryst}}$ was calculated for each peak position. The goodness of fit values $\boldsymbol{R}^2$ are listed for each fit of the Pseudo-Voigt function.

$\boldsymbol{\vartheta}_{\mathbf{Bragg}}$ / °	$\boldsymbol{\vartheta}_{\mathbf{Bragg,Ref}}$ / °	reflection	$\boldsymbol{d}_{\mathbf{cryst}}$ / nm	$\boldsymbol{R}^2$
15.05 ± 0.04	15.058	220	9.4 ± 0.7	0.7526
17.74 ± 0.01	17.736	311	9.8 ± 0.3	0.9474
21.57 ± 0.07	21.556	400	8.0 ± 0.9	0.4807
26.77 ± 0.16	26.743	422	6.7 ± 1.2	0.2281
28.58 ± 0.03	28.508	511	9.2 ± 0.8	0.6671
31.39 ± 0.02	31.305	440	10.3 ± 0.7	0.8269

Supplementary Materials S5

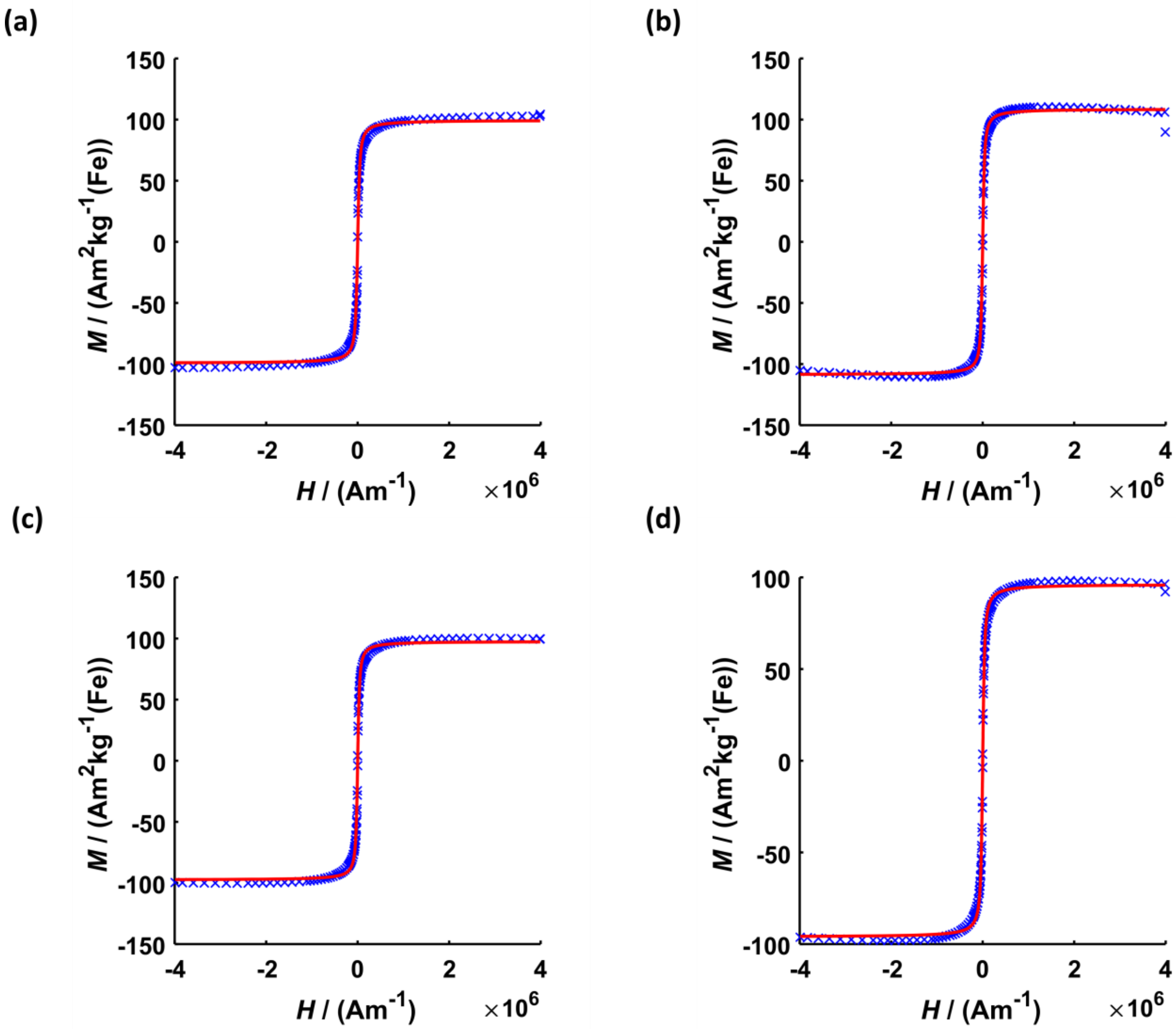

Figure S5: Hysteresis magnetization curve with a fit of the Langevin function (see equation (4)) for (a) dispersed MNP (R^2 = 0.99775), (b) PP@3%MNP (R^2 = 0.99887) (c) PP@5%MNP (R^2 = 0.99796) and (d) PP@7%MNP (R^2 = 0.99839).

Supplementary Materials S6

Table S6: SLP values of the hybrid stents. σ_{SLP} denotes the standard deviation.

Hybrid stent	SLP ± σ_{SLP}°
St@3%MNP	415 ± 12
St@5%MNP	346 ± 14
St@7%MNP	333 ± 9
